# Wear Resistance of Zirconia-Reinforced WC-6Co Hardmetals: A Case Study

**DOI:** 10.3390/ma19122436

**Published:** 2026-06-07

**Authors:** Boranbay Ratov, Volodymyr Mechnik, Edvin Hevorkian, Miroslaw Rucki, Daniel Pieniak, Zbigniew Siemiątkowski, Volodymyr Khomenko, Galiya Akhmedyanova

**Affiliations:** 1 Institute of Geology and Oil and Gas K. Turysova, Satbayev University, Satpaev Str. 22, Almaty 050013, Kazakhstan; ratov.bt@gmail.com; 2 V. Bakul Institute for Superhard Materials, National Academy of Science of Ukraine, Avtozavodska Str. 2, 04074 Kyiv, Ukraine; 3 Faculty of Production Engineering, University of Life Sciences in Lublin, 28 Głęboka St., 20-612 Lublin, Poland; 4 Institute of Mechanical Science, Vilnius Gediminas Technical University, 11 Sauletekio al., LT-10223 Vilnius, Lithuania; 5 Faculty of Safety Engineering and Civil Protection, Fire University, 52/54 Słowackiego Street, 01-629 Warsaw, Poland; dpieniak@apoz.edu.pl; 6 Faculty of Mechanical Engineering, Casimir Pulaski Radom University, Stasieckiego Str. 54, 26-600 Radom, Poland; 7 Institute of Nature Management, Dnipro University of Technology, Av. Dmytra Yavornytskoho 19, 49005 Dnipro, Ukraine; 8 Department of Mechanics and Oil and Gas Engineering, Faculty of Engineering, Toraighyrov University, Pavlodar 140008, Kazakhstan; akhmedyanova.g@teachers.tou.edu.kz

**Keywords:** composite, tungsten carbide, cobalt, zirconia, friction, wear resistance

## Abstract

WC–Co composites are widely applied in various industries due to their high hardness and wear resistance. The addition of zirconia further enhanced the composite, improving grain refinement and reducing the friction coefficient. Zirconia submicron particulate reinforcement strengthened the cobalt binder phase, significantly improving the wear resistance. Tribological tests were performed in ball-on-flat dry sliding mode, against an Al_2_O_3_ counter body. The specimen WC–6Co–10ZrO_2_ exhibited a decrease in volumetric loss Δ*V* by 66% compared to 94WC–6Co composite, and the wear rates *W_s_* showed a 50% decrease for 4 wt.% zirconia addition and up to 80% when 10 wt.% ZrO_2_ was added. In tribological tests, a five times larger load, 100 N instead of 20 N, caused an increase in the wear rate 1.1, 1.3, and 1.9 times for WC–Co, WC–6Co–4ZrO_2_, and WC–6Co–10ZrO_2_ compositions, respectively. Abrasive and adhesive wear mechanisms were identified.

## 1. Introduction

Hardmetals such as cemented carbides belong to a group of composite materials consisting of a carbide phase of high hardness bonded by a ductile metallic binder. These materials are widely applied due to their high hardness and wear resistance, and a suitability for harsh working conditions [1,2]. An important example of hardmetal is tungsten carbide with cobalt bond (WC–Co), which has been very popular since 1923. It is estimated that up to 72% of tungsten produced worldwide is used to produce WC carbide for fabrication of WC–Co composites [3]. Cobalt not only plays the role of a binder for hard particles, but it also provides toughness for the metal–matrix composite, so that the balance between hardness and toughness depends on the proportion and dispersion of cobalt phase in the composite. It was demonstrated in [4] that the Co phase contributed to the creation of numerous dimple fractures and, due to the inertia effect of crack propagation, improved plastic deformation of the composite.

For cutting tools application, the proportion of cobalt usually is from 6% up to 15% by mass [5]. The excellent balance of mechanical properties, including high fracture toughness and compressive strength, can be attributed to the low dihedral angle of the WC–Co system and cobalt’s ability to dissolve large amounts of tungsten carbide not dissolved in WC [6]. The performance of WC–Co hardmetals is dependent on several factors, such as cobalt binder proportion [7], initial powder morphology [8], carbide grain size [9], and microstructural homogeneity [10].

WC-based composites are used for components that are designed for use in harsh conditions, where excellent resistance to abrasive and erosive wear is required [11]. However, abrasive wear and brittle fracture as the main failure modes motivate research aimed at simultaneous improvement of the toughness and wear resistance [12]. Mechanical and tribological properties including wear of WC–Co composites have been systematically studied in search of ways to improve their performance [13]. Among others, understanding the mechanisms of WC-Co behavior under various mechanical loads and thermal conditions is critical for performance improvement of tools and components [14].

Generally, WC–Co composites are fabricated by powder metallurgy, WC and Co powders being first precisely mixed to ensure required homogeneity, then compacted and sintered [15]. WC–Co hardmetals are usually produced using liquid phase sintering techniques at temperatures below the melting points of both WC (2800 °C) and cobalt (1495 °C) [16]. In particular, it was demonstrated that dense and hard composites could be sintered using the Spark Plasma Sintering method (SPS) at temperatures 1100–1300 °C, at holding time varying from 5 up to 15 min [17]. In the present study, WC–Co hardmetal was sintered using a modified, patented SPS method to examine the possibility of improvement of its wear resistance.

Zirconia being itself an excellent engineering material with remarkable properties [18] can also serve as an additive for enhancement of WC-Co composites. Since zirconia coatings can be used as a thermal barrier to prevent oxidation [19], it can be expected, among others, that the presence of zirconia may reduce the intensity of WC and Co oxidation wear [20]. For instance, Jiand et al. [21] introduced ZrO_2_ additive to WC–12Co cermet and demonstrated a significant improvement in its wear resistance, especially at elevated temperatures. Wang with collaborators [22] published research results demonstrating the enhancing effects of zirconia addition on the microstructural features and related mechanical and tribological properties of WC-Co coatings. Analysis of pure WC and pure ZrO_2_ ceramics, as well as WC–Co, WC–Co–ZrO_2_, ZrO_2_–WC combinations, revealed good toughness and hardness of WC–Co–ZrO_2_ systems, and indicated promising perspectives of their wear resistance, worthy of further investigations [23]. Yang with colleagues [24] demonstrated that the addition of yttria and zirconia improved microstructure and mechanical properties of WC-8Co metal matrix composite, inhibiting the growth of tungsten carbide grains. Jiang with co-authors [25] reported that tetragonal ZrO_2_ inclusions exhibited good interfacial bonding with the Co matrix and WC filler, hindering intergranular fracture and enhancing toughness due to the stress-induced phenomenon of phase transformation.

However, wear resistance and related wear mechanisms of the zirconia-reinforced WC-Co composites have not been investigated. The aim of this paper is to report new results obtained from research on the WC–6 wt.%Co hardmetals reinforced with 3 wt.% yttria-stabilized zirconia (3YSZ) powders in proportions of 4 wt.% and 10 wt.% [26]. The addition of YSZ was demonstrated to enhance the metallic phase with hard, particulate reinforcement, to improve the binding of carbide grains, and to inhibit grain growth, thus improving the wear resistance of the composite.

## 2. Materials and Methods

The composites were fabricated using metallic cobalt powder of particle size 2.0–3.0 µm, tungsten carbide marked WC 1750 H with average particle size 2.0–4.0 µm (Global Tungsten & Powders spol. s.r.o., Bruntal, Czech Republic), and ZrO_2_ nanopowder partially stabilized with 3 wt.% Y_2_O_3_ (NANOE, Ballainvilliers, France) of average particle size 20–100 nm. Yttria served not only as a stabilizer for the zirconia phase, but also supported grain refinement, improving the overall uniformity of WC–Co carbide grains [27].

The powders were mixed in proportions 94 wt.%WC–6 wt.%Co (specimen WC–6Co), 90.24 wt.%WC–5.76 wt.%Co–4 wt.%ZrO_2_ (specimen WC–6Co–4ZrO_2_) and 84.6 wt.%WC–5.4 wt.%Co–10 wt.%ZrO_2_ (specimen WC–6Co–10ZrO_2_). After ultrasound dispersion in distilled water, the mixture was processed for 20 min with a Pulverisette 7 planetary micro mill (Fritsch GmbH, Idar-Oberstein, Germany) with balls’ diameters 1.5 mm. The rotational speed was 912 rpm, which corresponded with acceleration 640 m/s^2^. Then the mixed powders were dried in an oven for a period of 24 h at the temperature *T*_0_ = 80 °C.

The powders were sintered in graphite dies by the modified SPS method called electroconsolidation and described in another publication [28]. The cylindrical specimens had diameters 25 mm and height 5 mm. Based on the initial results published earlier [29], the following process parameters were chosen: vacuum 6 Pa, alternating current 5000 A, voltage 6 V, heating rate 500 °C/min, sintering temperature *T_sint_* = 1350 °C, uniaxial pressure *P* = 30 MPa, holding time *t* = 3 min. Electroconsolidation of WC–Co-based composites at these parameters had been found to ensure high density and small grain sizes of the specimens [26].

The as-prepared specimens were ground with diamond discs of the type 1A1-200 × 20 × 51 D213 (K50) G and polished for 3^1^/_3_ h using the polishing machine ATM Saphir 550 (ATM Qness GmbH, Mammelzen, Germany). The obtained surfaces were mirror-like and exhibited *Ra* below 0.1 μm, according to the requirements of relevant standard [30].

The microstructural features of the specimens were analyzed in a scanning electron microscope (SEM) Zeiss EVO 50 XVP (Carl Zeiss Microscopy GmbH, Jena, Germany) using morphological and material contrasts from Compact Zeiss BackScattered Detector (CZ BSD, Carl Zeiss Microscopy, Jena, Germany). The elemental composition and phase components were determined using energy dispersive X-ray spectroscopy (EDX) with application of an Ultim Max 100 analyzer (Oxford Instruments, Abingdon, UK). To verify the chemical composition of the phases visualized in Z-contrast, local energy-dispersive X-ray microanalysis was performed at four characteristic points of the structure. A field-emission scanning electron microscope type SU-70 EDS (Hitachi, Tokyo, Japan) was used to study the surface topography and to analyze the geometry of the wear traces after the tribotest. Photographs of the polished surfaces in polarized light were obtained from an optical microscope Axioscope 5 (Carl Zeiss Microscopy GmbH, Jena, Germany).

The friction coefficient *μ* and the specific wear rate *W_s_* were measured according to the ASTM [31] and ISO [32] standards using a friction testing machine produced by Optimol Instruments Prüftechnik GmbH (München, Germany). The reciprocating test mode with dry sliding of the ball-on-flat model was used with frequency *f* = 50 Hz. The tests were carried out with loads of 20 N and 100 N for *t* = 300 s. An Al_2_O_3_ ball of diameter 10 mm was used as the counter body. To study the geometry of the wear traces and the wear volume obtained as a result of the tribotest, a Taylor Hobson CCI interference profilometer (Taylor Hobson, Leicester, UK) was used. Each measurement was carried out on a 2.5 × 1.4 mm area, which allowed covering the entire wear trace and standardizing the measurements for each subsequent trace. The resulting 3D topography of the wear trace allowed calculating the wear volume. The diameter of the abrasion area on the counter body was measured using a Nikon MM-40/L3FA optical measuring microscope (Nikon Corp., Tokyo, Japan). The diameter was measured in two perpendicular directions, parallel to the course of the counter body and in the perpendicular direction, and then the arithmetic mean was calculated for each test made for a given material. The experiments were repeated three times, so that averages and deviations could be analyzed.

Based on the collected data on volumetric loss Δ*V* [mm^3^] and sliding distance *L* [m], specific wear rate *W_s_* was calculated using Equation (1) [33]:

(1) $$W_{s}=\frac{∆V}{F_{N}L},$$

where *F_N_* is the normal force applied to the counter body [N]. The sliding distance was calculated as follows:

*L* = 2*f*·*l*·*t*, (2)

where *l* is the single path of the reciprocating movement [m], *f* is the oscillation movement frequency [Hz], and *t* is the time [s].

## 3. Results and Discussion

The results presented in the following sub-sections cover microstructural features, friction coefficients, wear rates and wear mechanisms of the examined materials.

### 3.1. Microstructural Features

**Figure 1:**
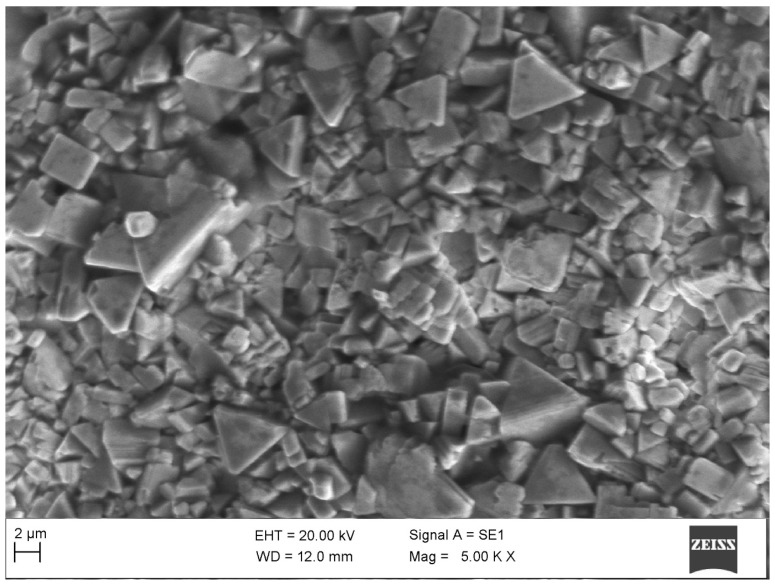
SEM image of the WC–6Co composite surface after sintering, before polishing.

Microstructural analysis of the surface of WC–6Co sintered samples demonstrated typical morphology of tungsten carbide grains seen in Figure 1. Since the surface had not been polished, the real morphology of the grains can be distinguished, which was formed during the liquid-phase sintering. The image clearly shows crystals of WC powder with pronounced faceting. The vast majority of grains have a shape of triangular prisms with sharp edges and corners. Such a clear geometric shape is an indicator of normal grain growth and high quality of the starting powder.

The structure shown in Figure 1 exhibited high polydispersity. Along with large grains (4–8 μm in size), a fraction of smaller crystallites (1–2 μm) is seen between the large grains. This corresponds to the particle size distribution of the initial powder (1–10 μm) and indicates dense packing of particles during the electroconsolidation process under uniaxial pressure *P* = 30 MPa. Since the content of the metal binding phase is small, ca 6 wt.%, no significant accumulations of free cobalt appeared on the surface. The cobalt bond is distributed mainly in the form of thin layers between the WC grains, enhancing their consolidation. Due to the absence of mechanical surface treatment, a high roughness and presence of deep shadows between groups of grains can be seen. The observed insignificant porosity is mainly represented by intergranular voids, typical for the free surface of a sintered composite.

**Figure 2:**
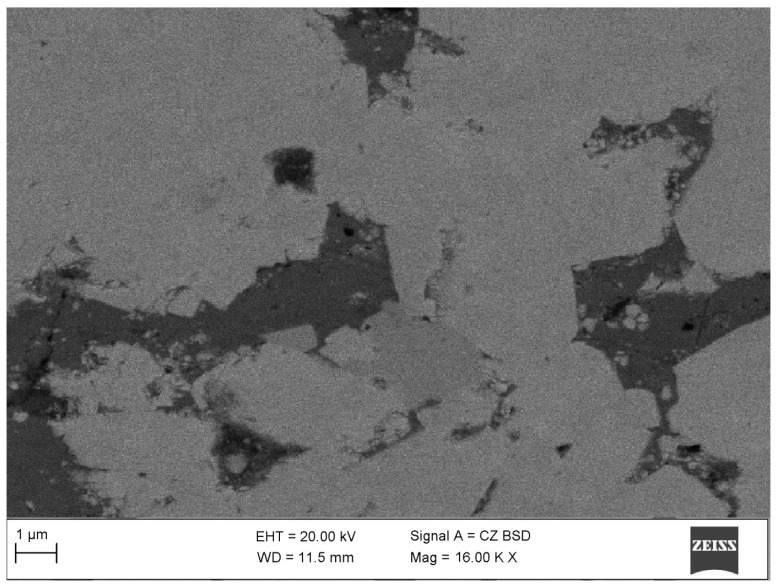
Microstructure of the polished WC–6Co–4ZrO_2_ composite surface (SEM image in the BSD mode).

Microstructure study on polished surfaces at high magnification in the contrast mode using Back Scattered Electrons Detector (BSED) allowed for identification of the distribution of reinforcing nanoparticles in the composite structure. Figure 2 obtained with Z-contrast presents the image with clearly distinguished main structural components. WC grains are visualized as the brightest areas due to the high atomic number of tungsten (Z = 74). They have clear boundaries and form a rigid skeleton of the material. The cobalt bond is represented in the form of darker areas between the carbide grains, since the atomic number of cobalt (Z = 27) is much lower than that of tungsten. Within the background of the dark cobalt matrix, some small, bright, dispersed inclusions of ZrO_2_ can be clearly seen. Their increased brightness compared to cobalt is explained by higher average atomic number of zirconium (Z = 40).

Image analysis reveals that the introduced ZrO_2_ nanoparticles of size 20–100 nm are located mainly inside the cobalt bond volumes, but they did not dissolve in Co during sintering. The zirconia particles are distributed relatively evenly, although in some places small agglomerates up to 500 nm can be found. In fact, such localization transforms the metallic bond into a particulate reinforced nanocomposite layer of Co + ZrO_2_. It is known that in metal alloy composites, specific wear rate decreases with the increasing percentage of ZrO_2_ particulate reinforcement dispersed along the grain boundaries [34]. The presence of hard, refractory oxide nanoparticles in the ductile cobalt phase can inhibit the dislocation motion and promote another dislocation–precipitate interaction known as Orowan and related mechanisms [35]. Apart from an increase in strength with no drop in ductility, the heat resistance of the material is improved. According to recent reports, the wear resistance of WC–Co system is critically dependent on the structure and hardness of the metal binder interlayers, because during service, the cobalt interlayers are worn out before the WC phase [36]. Thus, the improvement of wear performance can be expected in the WC–6Co–4ZrO_2_ composite.

Table 1 shows the results of roughness measurements after grinding and after the subsequent polishing process. The treatment resulted in similar surface roughness for samples of the studied materials. This table shows that without a ZrO_2_ addition, the surface of the WC–6Co composite was smooth after polishing. The root means square (RMS) value of surface roughness of this sample was *Ra* = 10 nm. The addition of ZrO_2_ in the amount of 4 wt.% and 10 wt.% to the WC–6Co composite was accompanied by an improvement of the surface roughness decreasing *Ra* down to 8 nm.

A significant decrease in the roughness *Ra* of the grinded surface after zirconia addition should be noted. The appearance of damage during the grinding process significantly reduces the fracture strength, fatigue strength, and wear resistance of ceramic components parts to be greatly reduced due to the stress concentration around grinding-induced surface and subsurface cracks [37]. Grinded in the same conditions, specimens with 4 wt.% zirconia exhibited higher resistance to the brittle damages and cracking than that of WC–6Co metal matrix composite, represented by a 24% lower *Ra*. Thus, it can be expected that the composites with zirconia additive will exhibit greater wear resistance.

The exceptional smoothness of the polished surfaces confirmed a high homogeneity and densification degree, which is extremely important for powder metallurgy. A high surface integrity is definitely a crucial aspect for composites designed to work in harsh conditions involving cyclic loads in severe applications [38].

### 3.2. Friction Coefficient and Wear Rate

**Figure 3:**
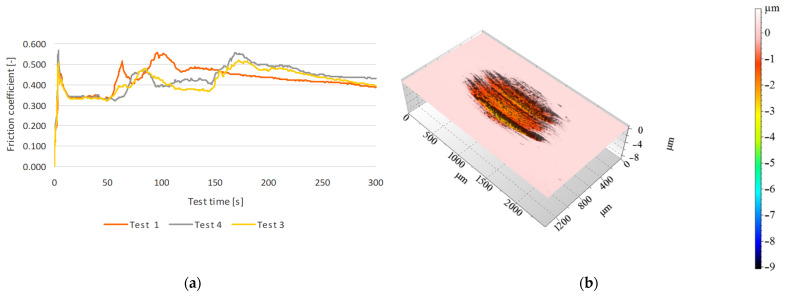
Wear test results for the WC–6Co sample against Al_2_O_3_ ball under load *F_N_* = 20 N: (**a**) Change in friction coefficient *μ* with test time *t*; (**b**) Topography of the worn surface.

**Figure 4:**
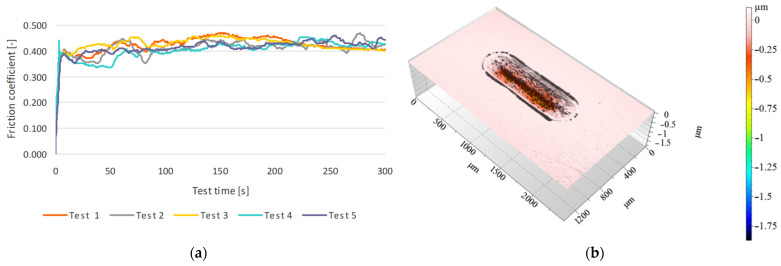
Wear test results for the WC–6Co–4ZrO_2_ composite against Al_2_O_3_ ball under load *F_N_* = 20 N: (**a**) Change in friction coefficient *μ* with test time *t*; (**b**) Topography of the worn surface.

**Figure 5:**
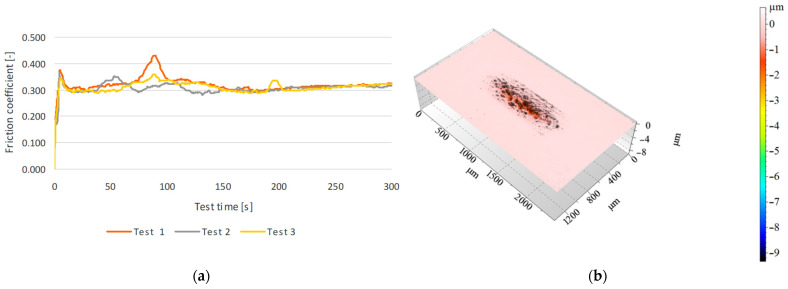
Wear test results for the WC–6Co–10ZrO_2_ material against Al_2_O_3_ ball under load *F_N_* = 20 N: (**a**) Change in friction coefficient *μ* with test time *t*; (**b**) Topography of the worn surface.

**Figure 6:**
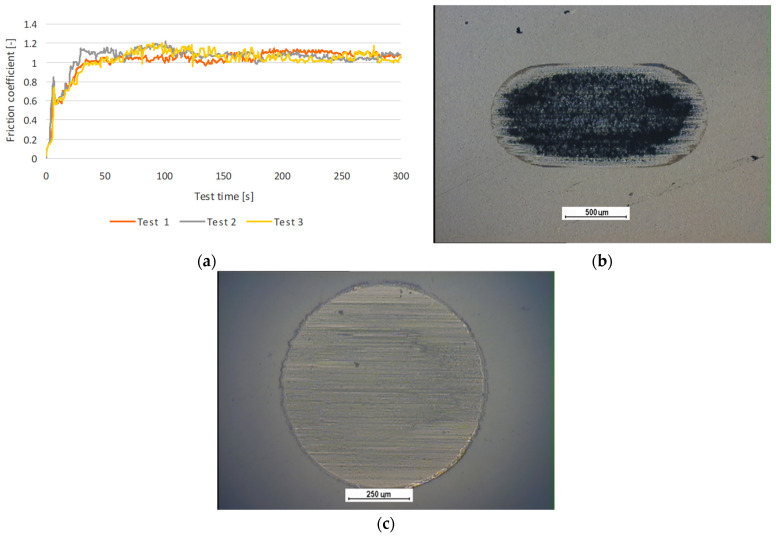
Wear test results for the WC–6Co sample against Al_2_O_3_ ball under load *F_N_* = 100 N: (**a**) Change in friction coefficient *μ* with test time *t*; (**b**) Photograph of the worn specimen’s surface; (**c**) Photograph of the worn surface of the Al_2_O_3_ ball counter body.

**Figure 7:**
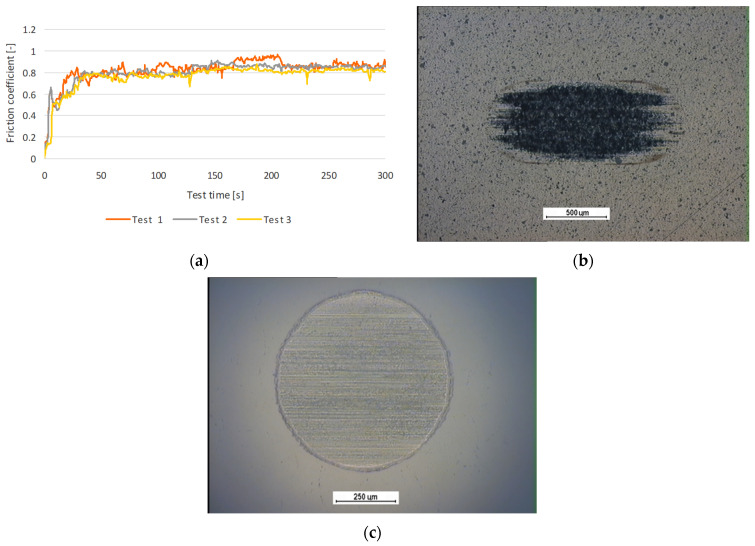
Wear test results for the WC–6Co–4ZrO_2_ sample against Al_2_O_3_ ball under load *F_N_* = 100 N: (**a**) Change in friction coefficient *μ* with test time *t*; (**b**) Photograph of the worn specimen’s surface; (**c**) Photograph of the worn surface of the Al_2_O_3_ ball counter body.

**Figure 8:**
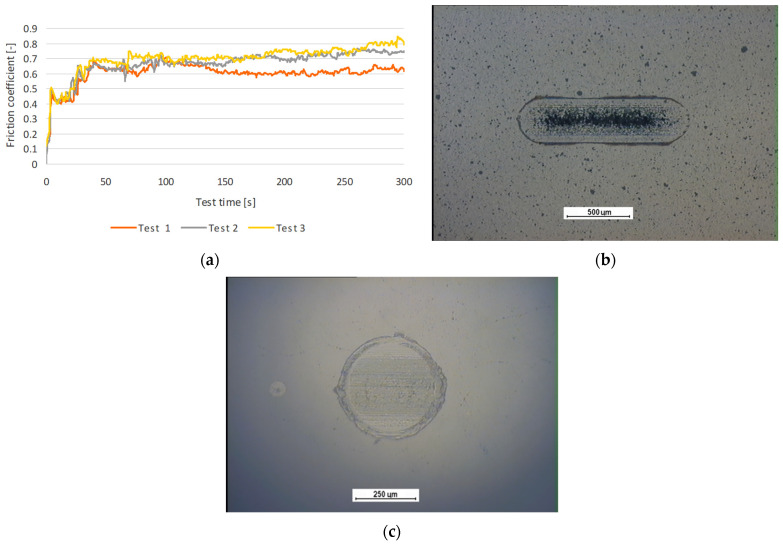
Wear test results for the WC–6Co–10ZrO_2_ sample against Al_2_O_3_ ball under load *F_N_* = 100 N: (**a**) Change in friction coefficient *μ* with test time *t*; (**b**) Photograph of the worn specimen’s surface; (**c**) Photograph of the worn surface of the Al_2_O_3_ ball counter body.

The results of tribological tests for two different loads, 20 N and 100 N, are given in Table 2 and Table 3, respectively. In all cases, the test lasted for *t* = 300 s, ensuring the sliding distance of *L* = 15 m. As a result, after the test, a worn area appeared in the tested specimen with its depth corresponding with the volume loss Δ*V*, and the worn ball formed a flattened side of a certain diameter dependent on the amount of the removed counter body material. The analysis shows that a five times increase in load caused a 94 times increase in the volume loss of the specimens and an increase in the worn area in the diameters ca. 2 times. In the tables below, the values of the friction coefficient μ are also given, measured for the test duration corresponding to the stabilized μ values. The diagrams of the friction coefficient for the three studied composites for loads of 20 and 100 N are shown in Figure 3, Figure 4 and Figure 5 and Figure 6, Figure 7 and Figure 8, respectively.

The diagrams of the friction coefficient *μ* in time *t* have maxima and regions of stabilized values. During the friction test, different wear mechanisms between the friction pairs dominated at different stages. From Figure 3a it is seen that the friction coefficient *μ* between the WC–6Co sample and Al_2_O_3_ ball exhibited maxima at different time points, reaching values of *μ* ≈ 0.56 at *t* ≈ 5 s and *t* ≈ 100 s, *μ* ≈ 0.51 at *t* ≈ 100 s, and *μ* ≈ 0.54 at *t* ≈ 170 s. After approximately 270 s of sliding, the friction coefficient *μ* decreased down to the level of ~0.42– 0.44 and remained almost constant.

The WC–6Co specimen exhibited significant dispersion for volumetric loss results (±0.482 × 10^−5^, mm^3^) and the maximum depth of the scratches seen in Figure 3b, which indicated the abrasive wear mechanism. This can be correlated with the irregular diagrams *μ* = *f*(*t*) where regions of almost constant value correspond with even wear, and local “jumps” of friction coefficient correspond with unsteady abrasive wear.

In contrast, the diagram of friction coefficient *μ* for the specimen with 4 wt.% zirconia shown in Figure 4a is much smoother, though exhibiting some fluctuations. After approximately 50 s of the test, the amplitude of the fluctuations became insignificant with average values between 0.37 and 0.42. The decrease in friction coefficient *μ* between the WC–6Co–4ZrO_2_ sample and Al_2_O_3_ compared to that of WC–6Co specimen can be attributed to an improved surface integrity. Moreover, the fact that a finer-grained homogeneous microstructure was formed after zirconia addition might have further contributed to the improvement in friction. In a recent report [39], it was noted that when ZrO_2_ was added to the WC–6Co composition, a more intense refinement took place of the phase components with a decrease in average lattice microdeformations ε*_c_* and ε*_a_* in directions *c* and *a*, respectively. This phenomenon obviously improved performance of the composite, exhibited also in a regular shape of the worn area shown in Figure 4b, where no single deep scratches are seen.

Among the tested materials, the lowest level of friction was recorded for the WC–6Co–10ZrO_2_ material in contact with Al_2_O_3_ ball counter body. In Figure 5a, the diagram exhibited no oscillations and a constant value around *μ* = 0.30 after 150 s of sliding test. In all tests, only one single maximum appeared that exceeded *μ* = 0.4. On the surface of the worn area shown in Figure 5b, smaller and more evenly scattered scratches were observed, accompanied with a decrease in volumetric wear compared to the specimens WC–6Co–4ZrO_2_ and WC–6Co.

Accordingly, the specimen WC–6Co–10ZrO_2_ exhibited a decrease in volumetric loss Δ*V* by 66% compared to WC–6Co composite, with the dispersion of results reduced down to ±0.107 × 10^−5^ mm^3^ (Table 1). The wear rates shown in Table 4 indicate wear resistance improvement with a 50% decrease in *W_s_* for 4 wt.% zirconia addition and 80% for 10 wt.% ZrO_2_ added, in comparison with WC–6Co.

Decrease in the specific wear rates *W_s_* of the WC-6Co composites with zirconia addition corresponds with the improved friction coefficient *µ* and smaller wear of both tested specimen (represented by volumetric loss Δ*V*) and Al_2_O_3_ ball counter body (represented by worn flat area diameter). The trend became even more prominent under the increased load *F_N_* = 100 N.

The results of the wear test under extreme load conditions *F_N_* = 100 N are shown in Figure 6, Figure 7 and Figure 8. The friction coefficient *μ* in the WC-6Co composite interaction with Al_2_O_3_ ball counter body exhibited extremely high values of 1.1–1.2 in all three test repetitions, as shown in Figure 6a. The initial running-in stage of the friction pair before stabilization was quite short, ca. 30–40 s. After that, *μ* remained between 1.0 and 1.2 with gradually decreasing amplitudes, exhibiting no sharp maxima. The respective images of worn areas of the WC-6Co specimen and Al_2_O_3_ ball are presented in Figure 6b,c.

Photographs of the worn area in Figure 6b exhibited deep and wide scratches on the bottom of the wear groove in WC–6Co sample. Tribological tests under a load of 100 N also led to intensive wear of the counter body. Quite deep parallel scratches were formed on the surface of the Al_2_O_3_ ball seen in Figure 6c, indicating intensive abrasive wear of the contacting pair under heavy load.

In the case of WC–6Co–4ZrO_2_ tested in the same conditions, the stabilized level of friction coefficient *μ* decreased by 25% compared to WC–6Co composite without zirconia additive, from ca. 1.1 down to ca. 0.8 shown in Figure 7a. The worn surfaces of both specimen and counter body in Figure 7b,c, respectively, exhibited scratches much smaller than those seen in Figure 6b,c. The role of abrasive wear evidently reduced in the case of WC–6Co–4ZrO_2_ composite compared to the WC–6Co material.

Even lower values of the friction coefficient *µ* = 0.6–0.7 were found in Figure 8a representing the results for WC–6Co–10ZrO_2_ composition. These can be attributed to the formation of a more homogeneous fine-grained microstructure. On the surface of the worn area of 10%YSZ-containing sample compared to 4%YSZ-containing one, smaller and more evenly distributed scratches are observed in the photograph in Figure 8b. A similar trend can be found on the flattened worn surface of the Al_2_O_3_ ball after the tribotest, shown in Figure 8c, compared to that seen in Figure 7c.

Specific wear rates *W_s_* for the studied compositions at an elevated load of *F_N_* = 100 N are given in Table 5. The wear rate for the WC–6Co composite appeared to be 11 times higher than the wear rate of the same sample tested at a load of *F_N_* = 20 N shown in Table 4. For comparison, recently reported indices of tolerance to abrasion damage 1/(*E*^2^*H*) [26] were added to Table 5. This index increased by 27% for the composite with 10 wt.% zirconia compared to WC–6Co material, reflecting a significant improvement of wear resistance.

The wear resistance of the WC–6Co composites fabricated by electroconsolidation significantly improved after zirconia additive was introduced. Compared to *W_s_* obtained for WC–6Co composite, the WC–6Co–4ZrO_2_ and WC–6Co–10ZrO_2_ samples exhibited wear rates lower by 40% and by 66%, respectively. This finding is especially important for those applications where the components are subject to heavy loads and friction forces.

Notably, the samples with zirconia addition used in this study exhibited a similar relative density 0.985, much higher than 0.948 for WC–6Co sample [40]. Even though WC–6Co had the highest hardness *HV*, its fracture toughness *K_IC_* and tolerance to abrasion damage 1/(*E*^2^*H*) appeared to be the lowest. The decrease in hardness after zirconia addition can be explained by the fact that *HV* of ZrO_2_ is by 30–35% lower than that of WC. However, the presence of zirconia enhanced interfacial areas, since its hardness was *HV* = 13–14 GPa, while that of cobalt was *HV* = 1.0–1.3 GPa. As a result, interfacial damages played a smaller role in the wear process.

### 3.3. Wear Mechanism

**Figure 9:**
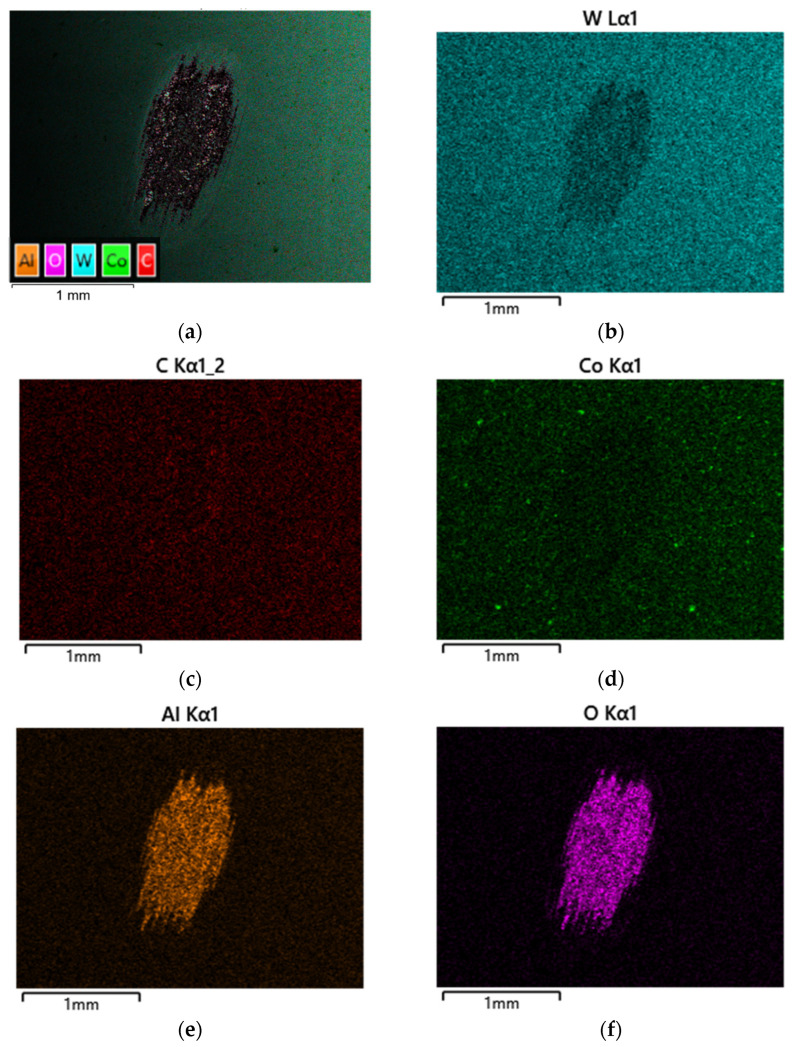
EDS results of elemental distribution on the worn surface of WC–6Co specimen: (**a**) Combined elemental map; (**b**) Tungsten distribution; (**c**) Carbon distribution; (**d**) Cobalt distribution; (**e**) Aluminum distribution; (**f**) Oxygen distribution.

It is difficult to identify the effect of a particular wear mechanism on the wear of a cemented carbide tool, but at least three mechanisms working together can be named: abrasive wear, diffusive wear, and adhesive wear [41]. To understand the mechanisms behind the improved wear resistance of the tested composites, elemental analysis of the worn surfaces was performed. In the element distribution maps seen in Figure 9, the area of the WC–6Co sample in contact with the Al_2_O_3_ counter body, apart from the basic elements W, Co, and C, also contained Al and O in some small localized areas. According to the EDS analysis, the distribution of WC and Co elements was uneven, in agreement with the uneven surface topography. Since the distribution of W, Co, and C on the sample surface outside the sliding path is uniform, it can be assumed that plastic deformation of the surface layer took place during the tribological test. The presence of Al from the counter body on the worn surface indicates that, in addition to abrasive wear, adhesive wear played important role in the degradation of WC–6Co sample.

It can be inferred from the analysis that abrasive wear accompanied by plastic deformation and subsequent failure was the primary wear mechanism of WC–6Co composite in interaction with Al_2_O_3_ counter body. Adhesive wear and intrusion of counter body debris into the tested composite surface took place, but had a lesser effect on wear intensity. Some of the oxygen seen in Figure 9f may originate from oxidation products [21] like WO_3_ and CoWO_4_, but since they are prone to be removed from the friction area during the wear process, their amount on the surface is rather insignificant.

**Figure 10:**
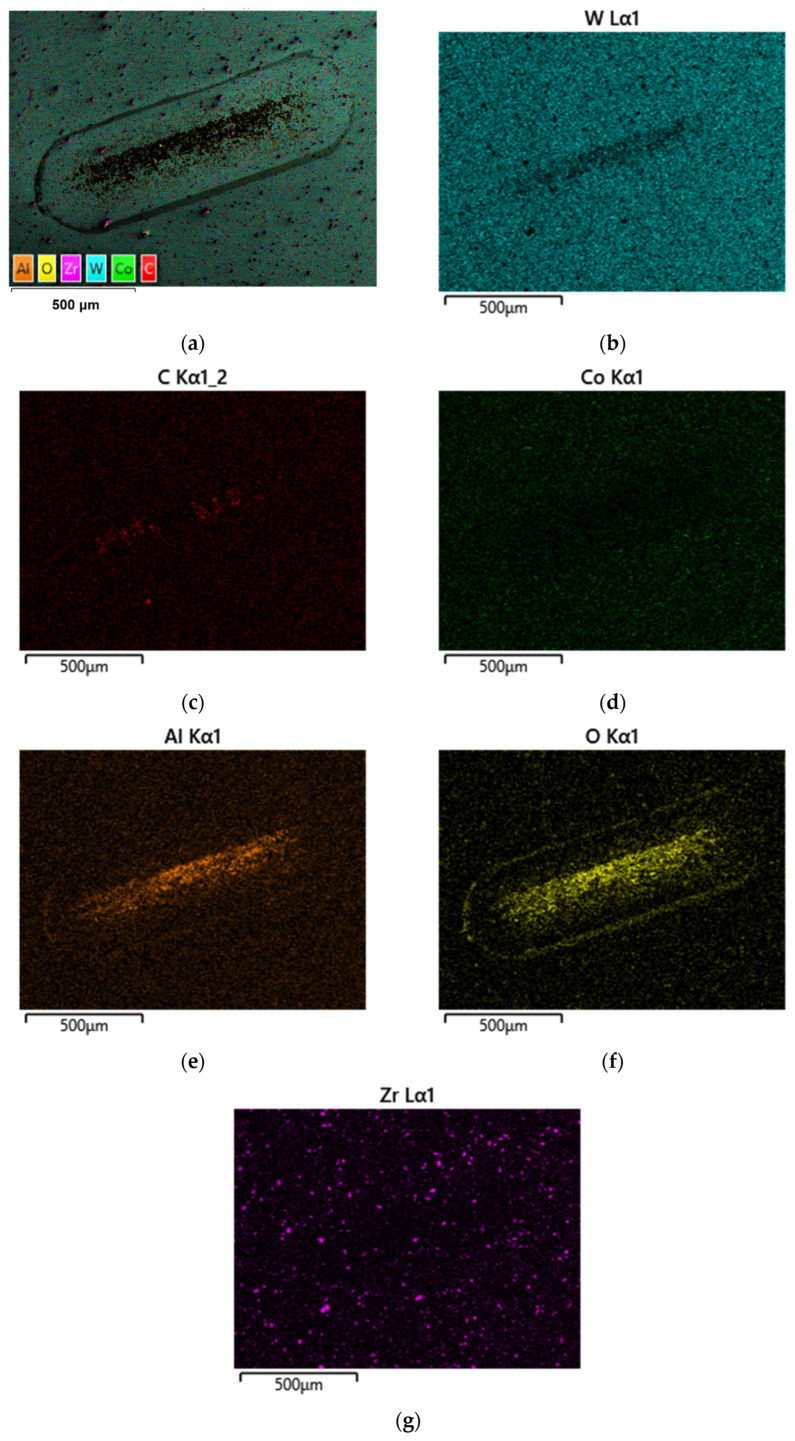
EDS results of elemental distribution on the worn surface of WC–6Co–4ZrO_2_ specimen: (**a**) Combined elemental map; (**b**) Tungsten distribution; (**c**) Carbon distribution; (**d**) Cobalt distribution; (**e**) Aluminum distribution; (**f**) Oxygen distribution; (**g**) Zirconium distribution.

In the elemental distribution maps of the 4%YSZ-containing composite worn surface, Al and O were also detected in addition to the basic elements W, Co, Zr, and C, as shown in Figure 10. This indicates presence of adhesive wear, like in the case of zirconia-free composite. However, the EDS analysis showed more uniform distribution of WC and Co elements on the worn area compared to WC–6Co specimen. The distribution uniformity after the tribological test explains the better surface integrity of WC–6Co–4ZrO_2_ composite compared to WC–6Co, represented by lower roughness after the same grinding process specified in Table 1 above.

It can be seen that the addition of ZrO_2_ to the WC–6Co composite reduced the intensity of adhesive wear. This phenomenon can be explained by the tetragonal structure of ZrO_2_ and formation of a finer-grained structure with a uniform distribution of phase components described in detail in [26]. In previous research [40] it was demonstrated that the composite WC–6Co without zirconia consisted of hexagonal WC phase (*a* = 0.2906 nm, *c* = 0.2837 nm), hexagonal graphite, and Co_3_W_3_C phase (*a* = 1.1112 nm), while after the addition of zirconia the composites consisted of WC and Co_3_W_3_C phases, amorphous carbon, and tetragonal ZrO_2_ (*a* = 0.36019 nm, *c* = 0.5174 nm).

**Figure 11:**
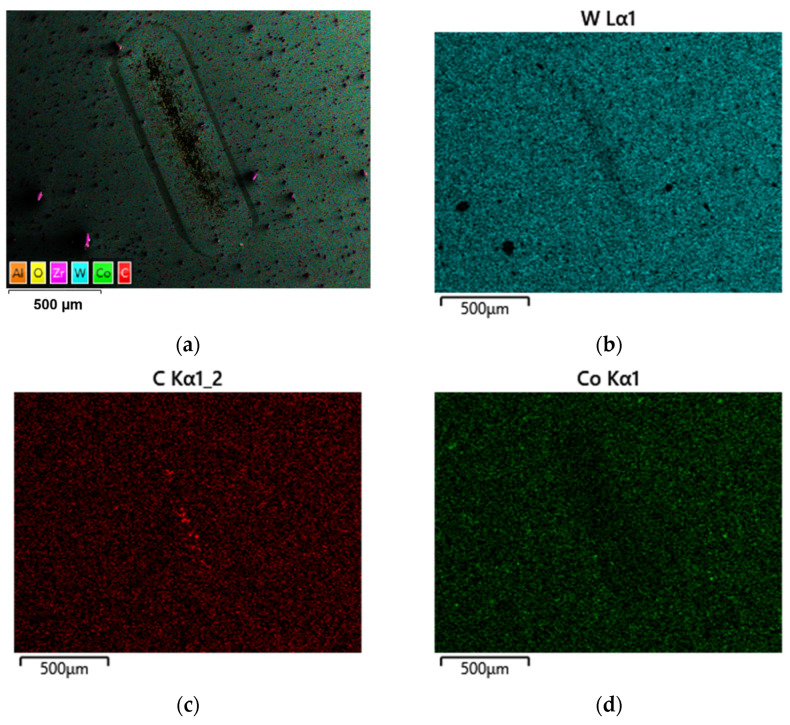

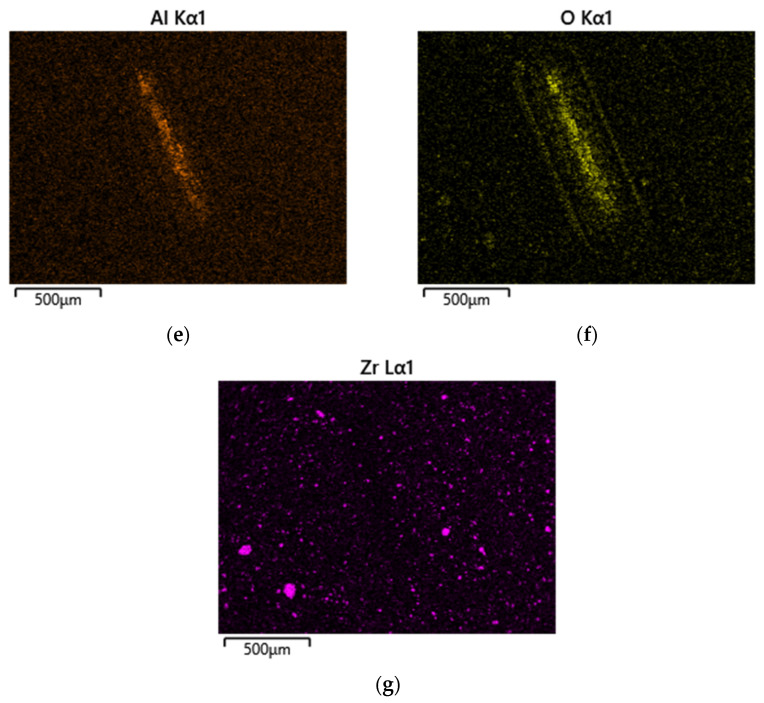
EDS results of elemental distribution on the worn surface of WC–6Co–10ZrO_2_ specimen: (**a**) Combined elemental map; (**b**) Tungsten distribution; (**c**) Carbon distribution; (**d**) Cobalt distribution; (**e**) Aluminum distribution; (**f**) Oxygen distribution; (**g**) Zirconium distribution.

The elemental distribution on the–10ZrO_2_ sample shown in Figure 11 is essentially the same as that of 4%YSZ-containing composite represented in Figure 10. However, traces of aluminum on the composite surfaces are smaller, and the plastic deformation area is smaller. In the zirconium map shown in Figure 11g, larger agglomerates can be seen than in the case of 4 wt.% of zirconia addition (Figure 10g). Presumably, zirconia concentrated in those agglomerates served as a dry lubricant reducing the friction coefficient and the adhesion component of the wear mechanism.

Thus, it was experimentally confirmed that the addition of 4 wt.%ZrO_2_ and 10 wt.%ZrO_2_ nanopowder to the WC–6Co metal matrix composite significantly improved its wear resistance, reducing the adhesive component of the wear mechanism and introducing a sort of dry lubrication. In some applications, e.g., in vacuum and low temperatures, adhesive wear is the main mechanism [42], so that zirconia additive can be particularly helpful in increasing the wear resistance of a tool.

The decreased friction coefficient contributed also to lower plastic deformation and reduced subsequent fatigue degradation of the composite. The presence of zirconia contributed to formation of fine-grained microstructure during the sintering process, and retention of submicron inclusions in the cobalt matrix made up advantages of a nanocomposite.

## 4. Conclusions

Microstructural analysis and tribological tests of 94 wt.%WC–6 wt.%Co metal matrix composite without and with addition of yttrium-stabilized zirconia in proportions 4 wt.% and 10 wt.% produced by electroconsolidation were carried out in this study and the following conclusions can be drawn.

Microstructural features and surface integrity appeared to be enhanced after zirconia addition. In particular, zirconia nanoparticles formed a nanocomposite in the cobalt matrix and introduced an additional toughening mechanism, while larger agglomerates worked as a dry lubricant in the tribological test. The improved tribological properties of WC–Co composites with ZrO_2_ additives can be attributed to a more uniform fine-grained structure and lower surface roughness.

The friction coefficient *μ* in contact with Al_2_O_3_ ball counter body under a load of 20 N oscillated in the range of 0.42–0.44 for WC–Co system, and for samples with 4 wt.% and 10 wt.% zirconia it was 0.35–0.42 and 0.30–0.32, respectively. The specific wear rate for the tested composites after the addition of zirconia dropped by 50% and 80%, respectively. Its values of order 10^−8^ mm^3^/(N·m) place the tested composites among highly wear-resistant materials.

An increase in the load by five times, from 20 to 100 N in the tribological test, led to an increase in the wear rate 1.1, 1.3, and 1.9 times for WC–Co, WC–6Co–4ZrO_2_, and WC–6Co–10ZrO_2_ compositions, respectively. It was found that the predominant wear mechanism for the 94WC–6Co sample under reciprocating friction with an Al_2_O_3_ ball was abrasive wear. Adhesive wear, resulting from the embedding of debris into the friction surface, had a lesser effect. The addition of ZrO_2_ to the WC–6Co composite suppressed adhesive wear, which can be explained by the tetragonal structure of ZrO_2_ and the formation of a finer-grained structure with a uniform distribution of phase components.

The results demonstrate the potential for developed compositions and the electroconsolidation technique in the production of composite materials for harsh working conditions, such as drilling hard and abrasive rocks.

## 5. Patents

Patent of Republic of Kazakhstan: Ratov, B.T.; Mechnik, V.A.; Hevorkian, E.S.; Bondarenko, N.A., Rucki, M.; Seidaliev, A.A.; Kuvanov, E.U.; Kalzhanova, A.B.; Morozov, D.; Samociuk, W. *Diamond-reinforced composite material*. No. 37698, Submission no. 2024/0909.1, 1 November 2024. Published 19 January 2025, Bulletin No. 51.

## Figures and Tables

**Table 1:** Results of roughness measurements.

Specimen	Roughness *Ra*, μm			
	After Grinding		After Polishing	
	Single Measurements	Mean (RMS)	Single Measurements	Mean (RMS)
WC–6Co	1.356	1.454	0.007	0.010
	1.294			
	1.516		0.010	
	1.661			
	1.399		0.013	
	1.501			
WC–6Co–4ZrO_2_	1.259	1.111	0.008	0.008
	1.024			
	1.087		0.007	
	1.179			
	0.982		0.009	
	1.138			
WC–6Co–10ZrO_2_	1.008	1.076	0.008	0.008
	1.081			
	1.294		0.010	
	1.122			
	0.975		0.008	
	0.977			

**Table 2:** Results of the wear tests under load *F_N_* = 20 N.

Specimen	Worn Flattened Surface Diameters, mm		Mean Diameter *d_m_* = (*d*_1_ + *d*_2_)/2, mm	Volumetric Loss of Specimen Δ*V* × 10^−5^, mm^3^	Average Friction Coefficient *μ*
	*d*_1_	*d*_2_			
WC–6Co	0.478	0.378	0.428	2.203 ± 0.482	0.47 ± 0.05
WC–6Co–4ZrO_2_	0.460	0.362	0.411	1.132 ± 0.271	0.42 ± 0.04
WC–6Co–10ZrO_2_	0.406	0.339	0.373	0.446 ± 0.107	0.30 ± 0.03

**Table 3:** Results of the wear tests under load *F_N_* = 100 N.

Specimen	Worn Flattened Surface Diameters, mm		Mean Diameter *d_m_* = (*d*_1_ + *d*_2_)/2, mm	Volumetric Loss of Specimen Δ*V* × 10^−5^, mm^3^	Average Friction Coefficient *μ*
	*d*_1_	*d*_2_			
WC–6Co	0.823	0.834	0.829	12.377 ± 1.238	1.108 ± 0.12
WC–6Co–4ZrO_2_	0.698	0.697	0.635	7.202 ± 0.714	0.800 ± 0.09
WC–6Co–10ZrO_2_	0.470	0.446	0.458	4.208 ± 0.421	0.650 ± 0.08

**Table 4:** Wear rates of the tested composites against Al_2_O_3_ ball counter body under load *F_N_* = 20 N.

Composition	Specific Wear Rate *W_s_* × 10^−8^, mm^3^/(N·m)
WC–6Co	7.344 ± 0.808
WC–6Co–4ZrO_2_	3.772 ± 0.339
WC–6Co–10ZrO_2_	1.487 ± 0.149

**Table 5:** Wear rates of the tested composites against Al_2_O_3_ ball counter body under load *F_N_* = 100 N and comparison to other properties.

Specimen	Specific Wear Rate *W_s_* × 10^−8^, mm^3^/(N·m)	Tolerance to Abrasion Damage 1/(*E*^2^*H*) [26]	Toughness *K_IC_*, MPa·m^0.5^ [40]	Hardness *HV*, GPa [40]
WC–6Co	8.252 ± 0.908	1.31 × 10^−16^	13.8 ± 0.71	15.9 ± 0.72
WC–6Co–4ZrO_2_	4.801 ± 0.432	1.40 × 10^−16^	16.9 ± 0.76	15.1 ± 0.33
WC–6Co–10ZrO_2_	2.805 ± 0.258	1.67 × 10^−16^	15.5 ± 0.60	13.4 ± 0.84

## Data Availability

The original contributions presented in this study are included in the article. Further inquiries can be directed to the corresponding author.

## Abbreviations

The following abbreviations are used in this manuscript:

BSED	Back Scattered Electrons Detector
EDX	Energy dispersive X-ray spectroscopy
RMS	Root means square
SEM	Scanning electron microscope
SPS	Spark Plasma Sintering
YSZ	Yttria-stabilized zirconia
